# Efficacy analysis of two surgical treatments for thoracic and lumbar intraspinal tumours

**DOI:** 10.1186/s12893-019-0602-9

**Published:** 2019-09-10

**Authors:** Zhaojun Song, Zhi Zhang, Yongjie Ye, Jiazhuang Zheng, Fandong Wang

**Affiliations:** Department of Orthopaedics, Suining Central Hospital, Suining, Sichuan People’s Republic of China

**Keywords:** Intraspinal tumour, Laminoplasty, Laminectomy, Ultrasonic bone curette

## Abstract

**Background:**

Surgery remains the main curative option for the treatment of intraspinal tumour. The purpose of the present study was to analyze the clinical outcomes of laminoplasty with process-lamina complex replantation compared with laminectomy with pedicle screw fixation for intraspinal tumours.

**Methods:**

In our retrospective analysis, 27 patients received tumour resection surgery by laminoplasty with reconstruction plate fixation and 32 patients received laminectomy with pedicle screw fixation. All patients were followed up for at least 1 year. Data, including surgical time, blood loss, volume of drainage, drainage time, hospital stay, complications, and neurological status were compared. In addition, imaging evaluation was also included.

**Results:**

Patients in the laminoplasty group had lower blood loss (laminoplasty group: 281.5 ± 130.2 mL; laminectomy group: 450.0 ± 224.3 mL; *p* = 0.001), shorter surgical time (laminoplasty group: 141.7 ± 26.2 min, laminectomy group: 175.3 ± 50.4 min; *p* = 0.003), lower volume of drainage (laminoplasty group: 1578.9 ± 821.7 mL, laminectomy group: 2621.2 ± 1351.0 mL; *p* = 0.001), shorter drainage time (laminoplasty group: 6.6 ± 2.5 days, laminectomy group: 9.7 ± 1.8 days; *p* = 0.000), and a shorter hospital stay (laminoplasty group: 16.9 ± 4.9 days, laminectomy group: 21.0 ± 4.4 days; *p* = 0.002) compared with patients in the laminectomy group. There were significant differences of oswestry dysfunction index (ODI) between the two groups at 12 months postoperatively (*p* = 0.034). The incidence of secondary spinal stenosis in the laminoplasty group was significantly reduced (*p* = 0.029).

**Conclusions:**

Laminoplasty in intraspinal tumour resection has a lower blood loss and volume of drainage, shorter surgical time and hospital stay as advantages over the standard laminectomy technique. Moreover, laminoplasty can effectively avoid iatrogenic spinal canal stenosis and thus enhancing functional recovery of spinal cord.

## Background

Complete tumour excision and keeping the integrity of spine are the two basic principles of surgical treatment [1–3]. Laminectomy with pedicle screw fixation is the most common surgical method for intraspinal tumour resection. However, this method destroys the posterior structure and stability of the spine, which results in scar adhesions, iatrogenic spinal stenosis, postoperative spinal deformity and pain, etc. [4, 5]. In recent years, laminoplasty has been considered to be more appropriate for patients with intraspinal tumours to avoid postoperative complications associated with laminectomy, such as refractory back pain and spinal deformity [6]. However, only a few high-level evidence-based studies have reported the comparative clinical effect from different perspectives between laminoplasty with reconstruction plate fixation and laminectomy with pedicle screw fixation for intraspinal tumours, and thus there remains considerable debate about the use of laminoplasty or laminectomy techniques for the treatment of intraspinal tumours. The purpose of the present study was to compare the clinical outcomes of laminoplasty with process-lamina complex replantation and laminectomy with pedicle screw fixation for intraspinal tumours.

## Methods

### Patients

A retrospective analysis was made to the two surgical treatments for intraspinal tumours of 59 cases which was approved by the Ethics Committee of the Suining Central Hospital. Fifty-nine patients with intraspinal tumours received tumour resection surgery in the spinal surgery centre of Suining Central Hospital. Twenty-seven patients received tumour resection surgery by laminoplasty with a reconstruction plate (Fuller Inc. Beijing, China) (defined as the laminoplasty group) and 32 patients received tumour resection surgery by laminectomy with pedicle screws (Trauson Inc., Jiangsu, China) (defined as the laminectomy group) between March 2015 and January 2018. Inclusive criteria: ① All cases of intraspinal tumors underwent operation for the first time. ② No spinal structure destruction or spine instability. ③ Clinical and pathological data were complete. ④ All patients were followed up for at least 1 year. Exclusion criteria: ① Facet joint was destroyed. ② Patient suffered from tumor recurrence. ③ Lesions lead to spinal destruction and instability.

There were 10 cases of meningioma, 9 cases of neurilemmoma, 3 cases of neurofibroma, 3 cases of lipoma and 2 cases of ependymoma in the laminoplasty group; there were 11 cases of neurilemmoma, 9 cases of neurofibroma, 5 cases of ependymoma, 4 cases of meningioma, 2 case of lipoma and 1 case of neuroepithelial cyst in the laminectomy group. The baseline characteristics of the two groups were not different, as shown in Table 1. Preoperative symptoms, neurological status on admission evaluated by American Spinal Injury Association impairment scale (ASIA) in both groups of patients are shown in Tables 2 and 3.

**Table 1:** Comparison of the baseline information of the two groups

Groups	Laminoplasty	Laminectomy	t/χ^2^ test	*P*-value
Male/female	12 / 15	15 / 17	0.035	1.000
Age (years)	53.3 ± 14.1	49.4 ± 13.8	1.076	0.286
BMI	24.7 ± 3.0	23.8 ± 4.1	−0.928	0.357
ODI	67.4 ± 3.6	68.6 ± 3.5	1.325	0.191

*BMI* Body Mass Index, *ODI* oswestry dysfunction index

**Table 2:** Clinical features of patients with intraspinal tumors in the laminoplasty group

Case no.	Gender	Age (years)	Symptom	Diagnosis	Position	ASIA grade	Replantation segments
1	1	70–79	motor and sensory disturbance	lipoma	T5–6	D	3
2	2	50–59	no	meningioma	T12	E	2
3	2	40–49	no	neurilemmoma	L2–3	E	2
4	2	80–89	motor disturbance	meningioma	T11–12	D	3
5	1	50–59	no	neurilemmoma	T5–8	E	5
6	2	60–69	motor and sensory disturbance	meningioma	T6	C	1
7	1	50–59	motor and sensory disturbance	neurilemmoma	T1	D	2
8	2	50–59	motor and sensory disturbance	neurilemmoma	L1	D	2
9	2	70–79	motor and sensory disturbance	meningioma	T2–3	C	3
10	1	40–49	motor and sensory disturbance	neurilemmoma	T4	D	2
11	2	70–79	motor and sensory disturbance	meningioma	T3	D	2
12	2	30–39	motor and sensory disturbance	meningioma	L1	D	2
13	2	50–59	motor and sensory disturbance	neurilemmoma	T12-L1	C	3
14	1	50–59	motor and sensory disturbance	meningioma	T1–2	C	2
15	1	40–49	sensory disturbance	lipoma	T12-L1	D	3
16	1	30–39	motor and sensory disturbance	neurofibroma	L3	C	2
17	2	20–29	motor and sensory disturbance	neurilemmoma	T10	D	2
18	2	30–39	motor and sensory disturbance	ependymoma	T12-L1	C	2
19	2	40–49	motor and sensory disturbance	meningioma	L1	D	2
20	1	40–49	motor and sensory disturbance	neurofibroma	T2	C	2
21	1	40–49	motor and sensory disturbance	neurilemmoma	T12	D	2
22	2	30–39	motor and sensory disturbance	lipoma	T12-L1	C	3
23	1	30–39	motor and sensory disturbance	meningioma	L2	D	2
24	1	50–59	motor and sensory disturbance	neurofibroma	L5	B	2
25	2	60–69	motor and sensory disturbance	neurilemmoma	T12-L1	C	2
26	2	60–69	sensory disturbance	ependymoma	L2	D	2
27	1	60–69	motor and sensory disturbance	meningioma	L3	D	2

*ASIA* American Spinal Injury Association impairment scale, *L* lumbar vertebra, *T* thoracic vertebra

**Table 3:** Clinical features of patients with intraspinal tumors in the laminectomy group

Case no.	Gender	Age (years)	Symptom	Diagnosis	Position	ASIA grade	Laminectomy segments
1	1	40–49	motor and sensory disturbance	neurilemmoma	T12-L1	D	3
2	2	30–39	sensory disturbance	ependymoma	L1–2	D	3
3	2	50–59	sensory disturbance	lipoma	T10-T11	D	2
4	1	60–69	motor and sensory disturbance	neurilemmoma	L2	D	2
5	2	10–19	no	ependymoma	L3	E	2
6	1	60–69	motor and sensory disturbance	neurilemmoma	T12	D	2
7	1	70–79	sensory disturbance	neurilemmoma	T5	D	2
8	2	50–59	sensory disturbance	neuroepithelial cyst	T10-L2	D	4
9	2	50–59	motor and sensory disturbance	neurofibroma	T5–6	D	3
10	2	50–59	motor and sensory disturbance	meningioma	T4	D	2
11	2	60–69	motor and sensory disturbance	meningioma	T4–5	D	2
12	1	50–59	motor and sensory disturbance	neurilemmoma	T11–12	D	3
13	2	40–49	sensory disturbance	neurofibroma	T9–10	D	2
14	1	50–59	motor and sensory disturbance	neurofibroma	L3–5	C	4
15	1	50–59	motor and sensory disturbance	neurilemmoma	T2	C	2
16	2	30–39	motor and sensory disturbance	neurofibroma	T11–12	B	3
17	2	30–39	motor and sensory disturbance	neurofibroma	T4	C	2
18	2	50–59	motor and sensory disturbance	ependymoma	T5	B	2
19	1	50–59	motor and sensory disturbance	neurilemmoma	L1	C	1
20	2	20–29	motor and sensory disturbance	meningioma	L1	D	2
21	2	30–39	motor and sensory disturbance	neurofibroma	T1–2	D	2
22	1	30–39	motor and sensory disturbance	neurilemmoma	T12-L1	C	3
23	1	60–69	motor and sensory disturbance	neurilemmoma	T10	D	2
24	1	60–69	motor and sensory disturbance	ependymoma	L3	C	1
25	1	50–59	motor and sensory disturbance	neurilemmoma	T12	C	2
26	2	40–49	motor and sensory disturbance	neurilemmoma	T12-L1	C	2
27	1	40–49	sensory disturbance	ependymoma	T12	D	2
28	2	50–59	motor and sensory disturbance	neurofibroma	T2	B	2
29	2	30–39	motor and sensory disturbance	neurofibroma	T11	C	2
30	1	30–39	sensory disturbance	lipoma	T11-L1	D	2
31	1	20–29	motor and sensory disturbance	neurofibroma	T6–7	B	2
32	2	60–69	motor and sensory disturbance	meningioma	T12	C	2

*ASIA* American Spinal Injury Association impairment scale, *L* lumbar vertebra, *T* thoracic vertebra

### Surgical technique

Preoperative examinations, including routine blood tests, blood biochemistry checks, blood electrolytes, coagulation evaluation, pretransfusion tests, blood type, chest X-ray, electrocardiogram, anterior posterior (A-P) and lateral plain films of the spine, and computed tomography (CT) and magnetic resonance imaging (MRI) of the spine, were applied to patients.

**Fig. 1:**
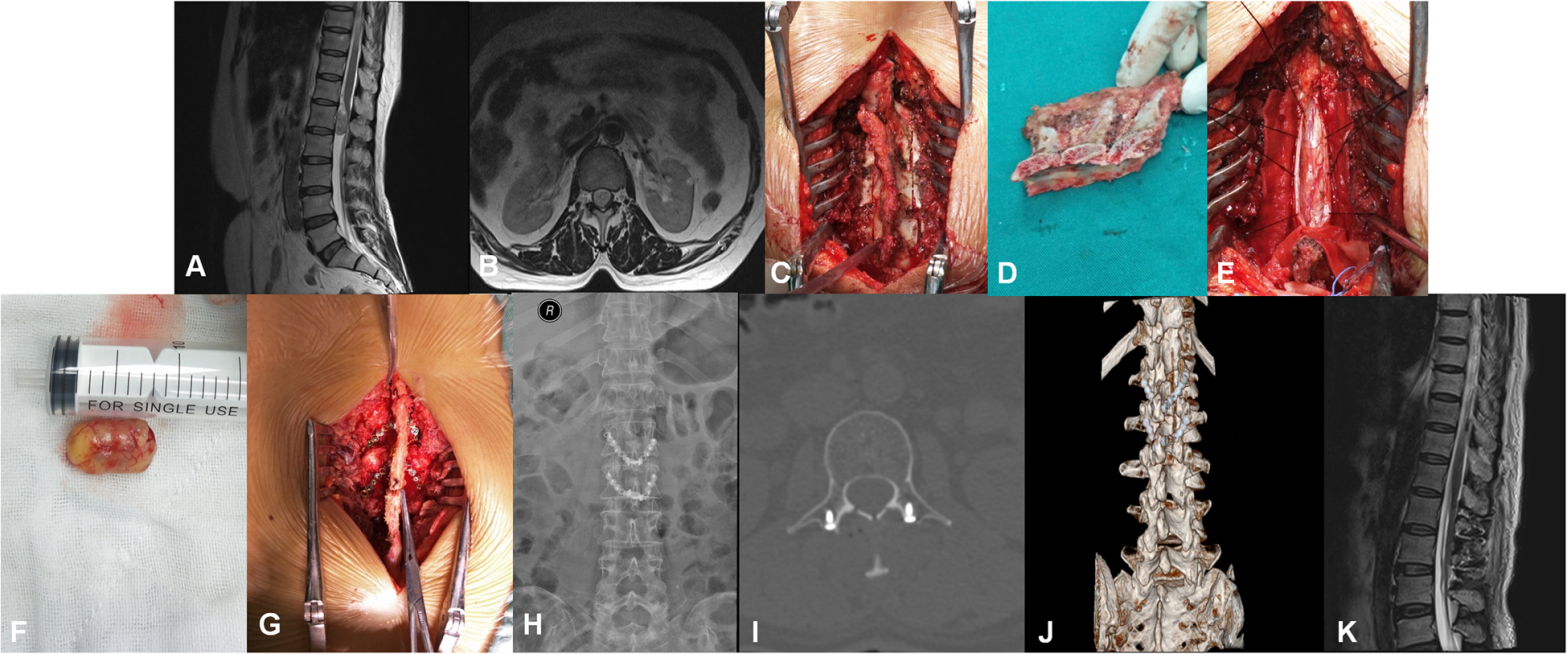
Case 8. Neurilemmoma at the L1 level in the laminoplasty group. **a** and **b** Preoperative MRI examination identified that the L1 spinal space was occupied by the tumour. **c** and **d** The excisional spinous process and vertebral plate complex. **e** and **f** The tumour was completely removed intraoperatively. **g** The excised spinous process and vertebral plate complex were fixed in situ with four reconstruction plates. **h** and **i** One week following the surgery, X-ray, computed tomography (CT) scan and **j** 3-D reconstruction examination indicated no fixation transposition or fracture. **k** Postoperative MRI showed the neurilemmoma was completely removed

Laminoplasty group (Fig. 1): Patients under general anaesthesia were placed in the prone position. The lesion segment of the vertebrae was determined by the C-arm. A posterior midline incision was made along the spine, centred on the tumour level. The paravertebral muscles were stripped to the medial border of the facet joint. The spinous process and laminar that required cutting were exposed according to tumour size. The lamina was cut with an ultrasonic osteotome between the lateral lamina and the inner facet joint. The supra- and interspinous ligaments, as well as the ligamentum flavum, were cut, and the spinous process and vertebral plate complex was separated and preserved. The tumour was exposed and excised, the spinous process and vertebral plate complex was reset, and the supraspinous ligament was sutured with thick silk thread. Two to four reshaping reconstruction plates were inserted and fixed bilaterally with 4 to 6 screws, following the resetting of the spinous process and vertebral plate complex. The in situ laminae replantation of the “viaduct” form was completed. Then, allogenic bone grafting was performed. Two drainage tubes were placed, and the incision was closed.

**Fig. 2:**
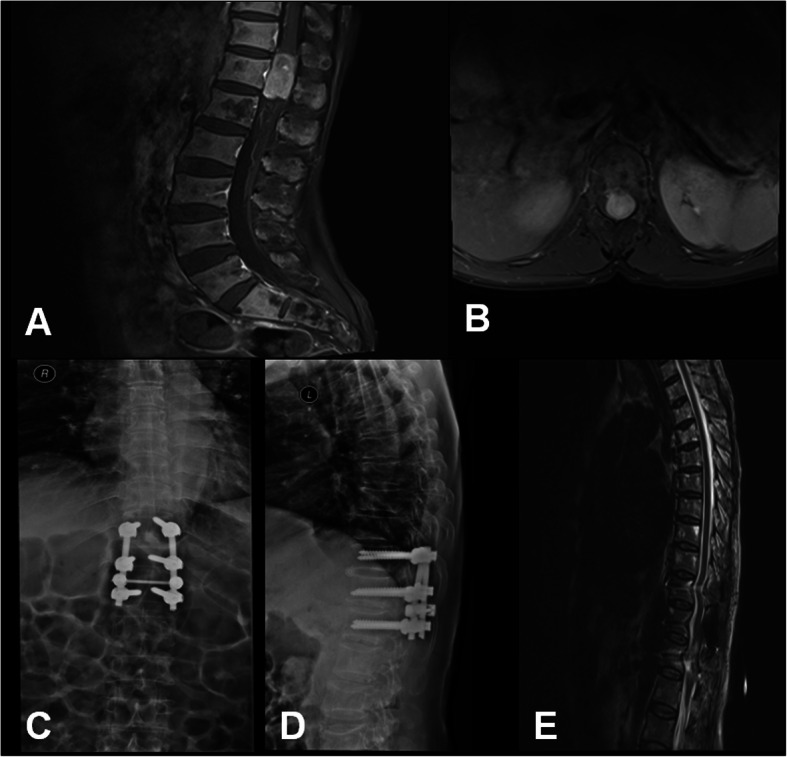
Case 6. Neurilemmoma at the T12 level in the laminectomy group. **a** and **b** Preoperative MRI examination identified that the T12 spinal space was occupied by the tumour. **c** and **d** One week following the surgery, X-ray examination indicated no fixation transposition or fracture. **e** Postoperative MRI showed the neurilemmoma was completely removed

Laminectomy group (Fig. 2): The spinous process and lamina were resected by bone rongeur with no laminoplasty, combined with pedicle screw fixation. The fixation segments were determined according to tumour size.

### Clinical evaluation

All patients were followed up for at least 1 year. Data, including surgical time, blood loss, volume of drainage, drainage time, hospital stay, and complications, were summarized, calculated and compared. Postoperative neurological status was evaluated by an independent surgeon using the ASIA classification (the neurological statuse recover to normal is classified as clinical success.), and patients were functionally assessed on the basis of the Oswestry disability index (ODI) at 3 and 12 months postoperatively. To determine the level of internal fixation and spinal stability, frontal and lateral X-ray films of the spine were performed at 1 week and 3, 6 and 12 months postoperatively and the changes in Cobb angle at surgical segment was measured (the Cobb angle greater than 10 degrees is classified as instability in surgical segment.). CT scans were performed to evaluate the bone growth of the replantation lamina at 3, 6 and 12 months postoperatively and the minimal cross-sectional area of vertebral canal in surgical site was measured. MRI was performed to detect tumour recurrence and scar oppression in the spinal canal and the repair of the ligaments at 6 months postoperatively.

### Statistical analysis

SPSS 19.0 statistical software (IBM, Armonk, NY, USA) was used for the statistical analysis and measurement data were recorded as the mean ± standard deviation (SD). An independent t-test or paired-samples t test was used to analyse the individual groups, and a Chi-square test was used to analyse enumeration data. A *p* value of less than 0.05 was considered statistically significant.

## Results

### Comparison of the surgical results

As shown in Table 4, patients in the laminoplasty group had lower blood loss (laminoplasty group: 281.5 ± 130.2 mL; laminectomy group: 450.0 ± 224.3 mL; *p* = 0.001), shorter surgical time (laminoplasty group: 141.7 ± 26.2 min, laminectomy group: 175.3 ± 50.4 min; *p* = 0.003), lower volume of drainage (laminoplasty group: 1578.9 ± 821.7 mL, laminectomy group: 2621.2 ± 1351.0 mL; *p* = 0.001), shorter drainage time (laminoplasty group: 6.6 ± 2.5 days, laminectomy group: 9.7 ± 1.8 days; *p* = 0.000), and a shorter hospital stay (laminoplasty group: 16.9 ± 4.9 days, laminectomy group: 21.0 ± 4.4 days; *p* = 0.002) compared with patients in the laminectomy group.

**Table 4:** The comparison of surgical results between the two groups

Groups	Surgical time (min)	Blood loss (ml)	volume of drainage (ml)	Drainage time (days)	Hospital stay (days)
Laminoplasty	141.7 ± 26.2	281.5 ± 130.2	1578.9 ± 821.7	6.6 ± 2.5	16.9 ± 4.9
Laminectomy	175.3 ± 50.4	450.0 ± 224.3	2621.2 ± 1351.0	9.7 ± 1.8	21.0 ± 4.4
t-test	−3.129	3.442	3.497	5.370	3.305
*P*-value	0.003	0.001	0.001	0.000	0.002

### Comparison of neurological statuses and ODI scores

Postoperative neurological status was evaluated by the ASIA classification and ODI. As shown in Table 5, there were no significant differences in improvement of neurological function between the two groups at 3 months postoperatively (*p* = 0.109), but there were significant differences in that at 12 months postoperatively (*p* = 0.036). As shown in Table 6, the ODI scores decreased over time and significantly improved compared to that before surgery in both groups, and there were significant differences of ODI between the two groups at 12 months postoperatively (*p* = 0.029, Paired-Samples T Test).

**Table 5:** Neurological statuses evaluated by ASIA classification

ASIA grade	A	B	C	D	E				
						Total	Total effective rate	Chi-square test	*P*-value
Before operation									
Laminectomy group	0	4	10	17	1	32		1.478	0.323
Laminoplasty group	0	1	9	14	3	27			
After 3 months									
Laminectomy group	0	2	5	15	10	32	31.3%(10/32)	2.576	0.109
Laminoplasty group	0	0	3	10	14	27	51.9%(14/ 27)		
After 12 months									
Laminectomy group	0	0	2	9	21	32	65.6%(21/32)	4.379	0.036
Laminoplasty group	0	0	0	3	24	27	88.9%(24/27)		

*ASIA* American Spinal Injury Association impairment scale

**Table 6:** Comparison of ODI scores between the two groups

Groups	Before operation	After 3 months	After 12 months
Laminoplasty	67.4 ± 3.6	45.8 ± 2.8	14.8 ± 3.2
Laminectomy	68.6 ± 3.5	46.7 ± 2.43	17.2 ± 4.8
Paired-Samples t-test	1.106	0.753	2.306
*P*-value	0.279	0.459	0.029

### Comparison between treatment effects and complications

Three patients suffered from complications in the laminoplasty group compared seven complications in the laminectomy group (*p* = 0.496, Pearson Chi-square). Early complications after laminoplasty occurred in 3 cases: 1 case with pulmonary infection, 1 case with urinary tract infection and 1 case with abdominal distension. By contrast, complications after laminectomy occurred in 7 cases: 2 cases with intracranial infections, 2 cases with pulmonary infection, 1 case with urinary tract infection, 1 case with hypoproteinemia and 1 case with deep vein thrombosis. Case no.4 and Case no.11 in the laminectomy group experienced intracranial infections and received intravenous anti-infection therapy and both patients were cured and discharged.

### Postoperative imaging evaluation

**Fig. 3:**
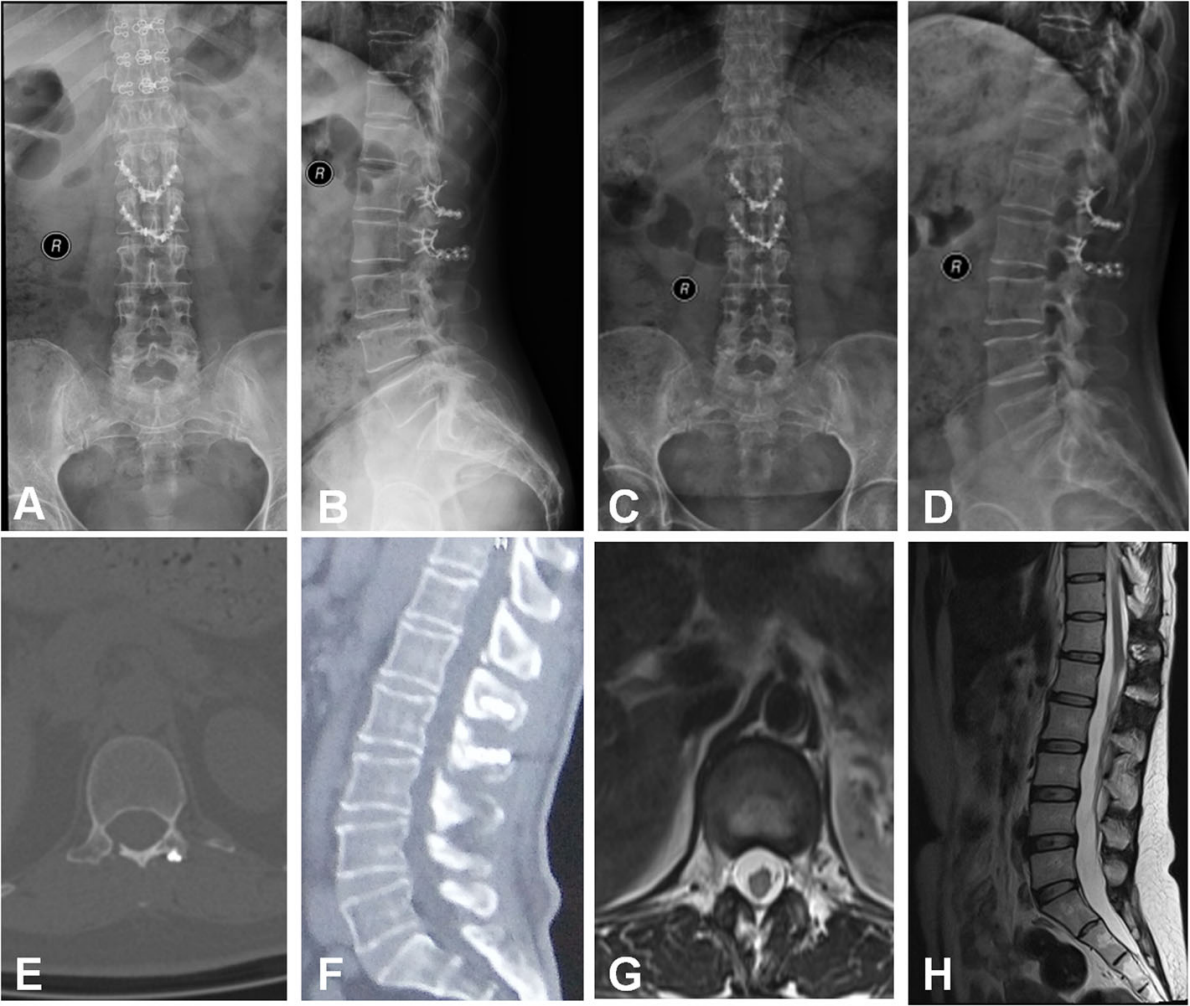
Patient with intraspinal tumour treated by laminoplasty with reconstruction plate fixation. **a** and **b** Six months following the surgery, X-ray examination indicated no fixation transposition or fracture, lumbar instability or kyphosis. **c** and **d** Twelve months following the surgery, X-ray examination indicated no fixation transposition or fracture, lumbar instability or kyphosis. **e** and **f** Six months following the surgery, CT scans indicated favourable osseous union of the replantation lamina. **g** and **h** Six months following the surgery, MRI indicated no tumour recurrence, and no intraspinal restenosis or scar adhesions

**Fig. 4:**
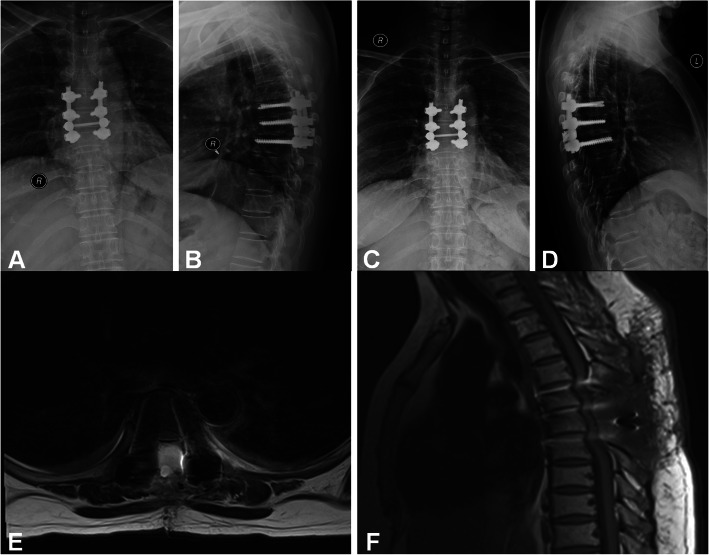
Patient with intraspinal tumour treated by laminectomy with pedicle screw fixation. **a** and **b** At 6 months following the surgery, X-ray examination indicated no fixation transposition or fracture, lumbar instability or kyphosis. **c** and **d** At 12 months following the surgery, X-ray examination indicated no fixation transposition or fracture, lumbar instability or kyphosis. **e** and **f** Twelve months following the surgery, MRI showed that the vertebral canal shape was irregular, and the dural sac was partially compressed

Postoperative imaging evaluation indicated no fixation transposition or fracture in both groups. The minimal cross-sectional area of vertebral canal in surgical site was (213.5 ± 17.1) mm^2^ in the laminoplasty group compared with (203.4 ± 17.3) mm^2^ in the laminectomy group (*p* = 0.029). The incidence of spinal instability was 1/27 in laminoplasty group compared with 5/32 in the laminectomy group according to Cobb angle (*p* = 0.224, Pearson Chi-square). In the laminoplasty group, CT scans indicated favourable osseous union of the replantation lamina approximately 3 to 6 months after surgery, while MRI indicated no tumour recurrence and no intraspinal restenosis or scar adhesions (Fig. 3). In the laminectomy group, MRI findings in a few patients demonstrated the iatrogenic spinal canal stenosis, a narrow vertebral canal, and the dural sac being partially compressed (Fig. 4).

## Discussion

Intraspinal tumours usually constrict the spinal cord and nerve root, which may lead to severe neurological deficits. Posterior laminectomy is usually used to remove these tumours, and the clinical effect in the short-term is good. Short-term follow-up studies on the laminectomy procedure have demonstrated a high degree of satisfaction [7]. However, this method destroys the posterior structure and stability of the spine, which results in scar adhesions, iatrogenic spinal stenosis, postoperative spinal deformity and pain, as well as a high risk of peridural adhesions and spinal cord injury [4, 5, 8].

At present, increasing numbers of neurosurgeons are aware of the importance of reconstruction of spinal stability and reducing postoperative complications for postoperative recovery and quality of patients, and pay great attention to the maintenance of spinal stability during the operation. Retaining the posterior column complex requires retaining the majority of the posterior column of the spine, the attachment of the sacrospinal muscle is preserved, and the posterior dynamic stabilization structure is maintained. As a result, the stability of the spine is well preserved [9]. The reconstruction of the posterior bone structure of the spine provides an important physiological condition for maintaining the skeletal structure of the spine after laminoplasty. A study by Zhou demonstrated that the in situ replantation of the spinous process and vertebral plate complex with plate fixation was able to improve spinal stability, compressive resistance and anti-bending, −shearing and -rotation abilities [10]. In addition, the supra- and interspinal ligaments are important structures for spinal flexion stability. Many scholars have proposed reconstruction of the supra- and interspinous ligaments [11]. In our study, the supraspinous ligament was repaired with thick silk sutures after resection of the tumour and the in situ replantation of the spinous process and vertebral plate complex. There is an increased risk of durotomy and neural injury, especially when performing osteotomy in a deep area, in a narrow surgical field, or in a revision situation [12]. Beyond that, it has been shown that laminectomy may cause more instability due to damage of posterior elements of the spinal column, which may induce subsequent kyphosis [9].

The osseous spinal canal is restored after replantation of the spinous process and vertebral plate complex, which reduces the risk of compression and re-injury of the spinal cord and iatrogenic spinal canal stenosis. In the laminoplasty group, the minimal cross-sectional area of vertebral canal in surgical site was (213.5 ± 17.1) mm^2^ compared with (203.4 ± 17.3) mm^2^ in the laminectomy group (*p* = 0.029), and CT and MRI findings in a few patients demonstrated some bone tissue entering into the vertebral canal, an irregular vertebral canal, and the dural sac being partially compressed. There were perhaps many reasons for the iatrogenic spinal canal stenosis. Bone regrowth around the dura may reproduce pathological conditions which was reported by Guigui P and Levy WJ [13, 14]. Shimazaki K reported that recurrence of spinal stenosis and claudication after laminectomy due to an ossified extradural pseudocyst [15]. A study by Mearini demonstrated that local pain was higher and recovery time was longer in patients with laminectomy [16]. Laminoplasty can reduce the occurrence of these conditions [14]. In our study, 88.9% of the patients obtained good condition (ASIA grade E) compared with 65.6% in the laminectomy group, and the laminoplasty group was better in neurological outcome and ODI at 12 months postoperatively, which is similar to that reported by Wen JP and Sandalcioglu IE [17, 18]. This might be related to the fact that laminoplasty can effectively avoid iatrogenic spinal canal stenosis.

The use of ultrasonic vibration for the cutting of bone has been widely adopted in the surgical field in recent years which can decrease the risk of damage to surrounding soft tissues and critical structures such as nerves and vessels, especially during osteotomy procedures [19]. Sanborn et al. reported that there is a notable reduction in osseous bleeding in ultrasonic osteotomy, which may be attributable to a local haemostatic effect due to thermal and cavitation effects [20]. The total drainage amount and drainage time in the laminectomy group were greater than that in the laminoplasty group (*p* < 0.05). The in situ replantation of the spinous process and vertebral plate complex places the dural incision close to the inner surface of the lamina, which finally reduces cerebrospinal fluid leakage.

The complication rate were 11.1% in the laminoplasty group compared with 21.9% in the laminectomy group, which is similar to that reported by Patil CG [21]. Pulmonary infection was the most common complication. There were three patients suffered from pulmonary infection, ages 81, 77 and 64. Airway management is key to preventing pulmonary infection, especially for elderly patients. Intracranial infection is the dangerous complication, a previous study revealed that 0.4% of patients were complicated by intracranial infection after after spinal operation [22]. Two patients in the laminectomy group experienced intracranial infections and received intravenous anti-infection therapy and both patients were cured and discharged. Surgical procedures, internal fixation, and cerebrospinal fluid leak were associated with an increased incidence of intracranial infection [23]. Once intracranial infection happens, the major treating measures are thorough debridement and intravenous anti-infection therapy with adequate dosage and duration [24].

There are several limitations of the current study, including a small sample size and relatively short-term follow-up period. Overall, laminoplasty in intraspinal tumour resection has a lower blood loss and volume of drainage, shorter surgical time and hospital stay as advantages over the standard laminectomy technique. Moreover, laminoplasty can effectively avoid iatrogenic spinal canal stenosis and thus enhancing functional recovery of spinal cord.

## Conclusions

Laminoplasty in intraspinal tumour resection has a lower blood loss and volume of drainage, shorter surgical time and hospital stay as advantages over the standard laminectomy technique. Moreover, laminoplasty can effectively avoid iatrogenic spinal canal stenosis and thus enhancing functional recovery of spinal cord.

## Data Availability

The datasets used and/or analysed in the current study are available from the corresponding author on reasonable request.
